# Analysis and curative effect of ocular ischemic diseases caused by carotid artery stenosis

**DOI:** 10.3892/etm.2013.979

**Published:** 2013-02-27

**Authors:** YITAO YAN, XINLI ZHANG, YUXIN YANG, LIYING HAN, HUIMIN WANG, JUNXI HU

**Affiliations:** 1 Department of Ophthalmology, The First Affiliated Hospital of Xinxiang Medical University, Weihui, Henan 453100;; 2 Department of Ophthalmology, The Second People’s Hospital of Xinxiang, Xinxiang, Henan 453000;; 3 Department of Ophthalmology, Nanyang Eye Hospital, Nanyang, Henan 473014, P.R. China

**Keywords:** ocular ischemia, carotid artery stenosis, curative effect

## Abstract

Carotid artery stenosis is a notable cause of ocular ischemic disease. To analyze the ocular ischemic diseases caused by carotid artery stenosis and to seek effective treatment, a retrospective review of 182 patients with carotid artery stenosis was performed. These patients were administered medical treatment, carotid artery stenting (CAS) or carotid endarterectomy (CEA) and the curative effects of the three different treatments were compared. The results demonstrated that all three treatments helped to improve the disease. Both carotid endarterectomy and carotid artery stenting were more effective than medical treatment (P<0.05). There was no significant difference between carotid endarterectomy and carotid artery stenting (P>0.05). In conclusion, timely diagnosis and suitable treatment for ocular ischemic diseases caused by carotid artery stenosis are necessary due to the complicated clinical manifestation. This study suggested that carotid endarterectomy and carotid artery stenting are effective techniques that may relieve this disease.

## Introduction

Carotid artery stenosis is one of the most common neurological diseases. Depending on the severity of this disease, patients show a number of brain and/or ocular ischemic symptoms and signs (1). Considering its high occurrence rate in neurology and its correlation with risk factors such as gender, hypertension and smoking, ocular ischemic diseases caused by carotid artery stenosis are generally recognized as a neurological disease (2,3). Complex and diverse clinical manifestations of the disease may, however, lead to a missed diagnosis or misdiagnosis. Some patients may then develop amaurosis fugax, diplopia, decreased vision or permanent blindness. Doctors must therefore analyze conditions thoroughly to offer timely and effective treatments.

Some scholars (1) specifically defined ‘ocular ischemic syndrome’ as a series of brain and eye clinical syndromes caused by chronic severe carotid artery obstruction or stenosis resulting in brain and eye insufficiency. However, in this study, a generalized concept was applied and all ocular ischemic diseases related with carotid artery obstruction or stenosis were analyzed, including ocular ischemic syndrome (according to definition), retinal central/branch vein occlusion, external ophthalmoplegia, ischemic optic neuropathy and neovascular glaucoma. By retrospective analysis of patients with carotid artery stenosis who were diagnosed at the First Affiliated Hospital of Xinxiang Medical University in recent years, the related etiological factors, clinical presentations and treatment effects were investigated.

## Subjects and methods

### Subjects

The clinical data were gathered from 182 patients with carotid artery stenosis who were diagnosed in the ophthalmology, neurology, neurosurgery or intervention departments of the First Affiliated Hospital of Xinxiang Medical University (Weihui, China) from January 2002 to November 2009. Among them, 107 were males and 75 were females aged between 24 and 87 years (mean, 65.24±8.65 years). All patients were diagnosed using carotid color Doppler or digital subtraction angiography (DSA) to exclude the possibility of various primary eye diseases, such as primary glaucoma, iridocyclitis, retinal vein occlusion, high myopia, retinal pigment degeneration, retinochoroiditis, diabetic retinopathy or congenital fundus anomaly. This study was conducted in accordance with the declaration of Helsinki, and with the approval of the Ethics Committee of the First Affiliated Hospital of Xinxiang Medical University. Written informed consent was obtained from all participants.

### Patients’ general information

The clinical data of the patients were gathered for retrospective analysis. The data included general characteristics of patients, body and eye medical histories (including amaurosis fugax and eye pain), past medical histories (including hypertension, diabetes, hyperlipidemia and coronary heart disease), personal histories (including smoking and drinking), the department where the patient was first treated, detailed eye symptoms and treatment procedures. The 182 patients were divided into two groups; 78 cases with ocular ischemic symptoms were classified as group A and 104 cases without ocular ischemic symptoms were classified as group B.

The 78 cases in group A were treated with drugs (including antihypertensives, antihyperglycemic agents, antihyperlipidemic agents or anticoagulants in a total of 19 cases), carotid artery stenting (26 cases) or carotid endarterectomy (33 cases). Certain patients with ocular ischemic symptoms also received eye treatments concomitantly (such as retinal laser photocoagulation, drugs to lower intraocular pressure or cyclocryotherapy).

### Evaluation criteria

The position and degree of the carotid artery stenosis were judged according to the results of the angiography. The status of the ophthalmic artery hemodynamics was analyzed using color Doppler flow imaging (Philips HD7). The detection frequency was 10 MHz and various ophthalmic artery hemodynamic indices were measured, including peak systolic velocity (Psv), end diastolic velocity (Edv), mean glow velocity (Vm), resistive index (RI) and pulsatility index (PI). Each index was measured in triplicate and the mean value was used for further analysis.

The effects of different treatments on group A patients were evaluated according to the following scale: i) Significantly effective; the patient felt that eye condition improved markedly. Vision of patients with decreased visual acuity was improved at least 2 lines after treatment, or ophthalmic artery hemodynamic indices were obviously improved; ii) Improved; the patient felt that eye status remained stable or had mild improvement. Vision of patients with decreased visual acuity was improved 1 line or remained stable after treatment, or ophthalmic artery hemodynamic indices showed some improvement; iii) Invalid; the patient felt symptoms were worse or vision decreased or ophthalmic artery hemodynamic indices were notably decreased; iv) Uncertain; the patient had occasional ocular ischemic symptoms or had no ophthalmic artery hemodynamic indices.

### Statistical analysis

Statistical data were presented as means ± SD or a percentage. The data were analyzed using SPSS 14.0 software (SPSS Inc., Chicago, IL, USA) for the t-test and χ^2^ test to calculate whether the two groups were statistically different. To avoid confounding and interaction between disease-related factors, one-way ANOVA and unconditional logistic regression were performed to analyze the correlation between various factors and onsets of the two groups of patients. P<0.05 was considered to indicate a statistically significant result.

## Results

### Comparison of patient age and gender

Among 182 patients, 19 were aged <50, 35 were aged 51 to 60, 68 were aged 61 to 70, 45 were aged 71 to 80, and 15 were aged >80. The average age of group A (64.41±9.45) was a little higher than that of group B (62.12±11.32) but there was no significant statistical difference (t=0.754, P>0.05).

### The position and degree of carotid artery stenosis

Carotid artery stenosis of both group A and B patients was mainly located in the common carotid artery bifurcation and internal carotid artery inlet. In group A, 75.64% (59) of cases had an over half diameter reduction and the degree of stenosis was 43–100% (mean, 69.13±7.46). The degree of stenosis in group B was 34–100% (mean, 48.34±9.23). There was a significant difference between the degree of the two groups (t=0.754, P>0.05).

### Risk factors associated with carotid artery stenosis

Group A consisted of 43 males and 35 females, including 46 patients with hypertension, 41 with hyperlipidemia and 35 smokers; group B consisted of 59 males and 45 females, including 53 patients with hypertension, 48 with hyperlipidemia and 40 smokers. According to the results of one-way ANOVA and unconditional logistic regression, the onset of carotid artery stenosis was closely associated with gender (male), hypertension, hyperlipidemia and smoking (χ^2^=4.562, 5.151, 4.471 and 4.463, respectively; P<0.05).

### Distribution of first-visit patients

Among the 78 patients with ocular ischemic symptoms (group A), 39 were first seen in the Neurology department (20 of them transferred to interventional treatment later) and 31 were first seen in Neurosurgery (4 of them transferred to interventional treatment later). Only 8 patients were first seen in Ophthalmology (2 of them transferred to Neurosurgery and 2 patients transferred to interventional treatment later).

### Incidence of ocular ischemic diseases

In addition to investigating ophthalmic medical records (including retinal hemorrhage, edema, visual field defects and increased intraocular pressure), discharged patients were followed up. Patients of group A recalled several symptoms, such as amaurosis fugax, eye swelling and periorbital pains (ischemia of ocular anterior segment) or diplopia. The frequencies of ocular ischemic symptoms are shown in Table I.

### Composition of ocular ischemic diseases

After classifying cases according to final diagnoses, 31 patients in group A had ocular ischemic diseases. The composition is shown in Table II.

### Ophthalmology treatments

**Figure 1:**
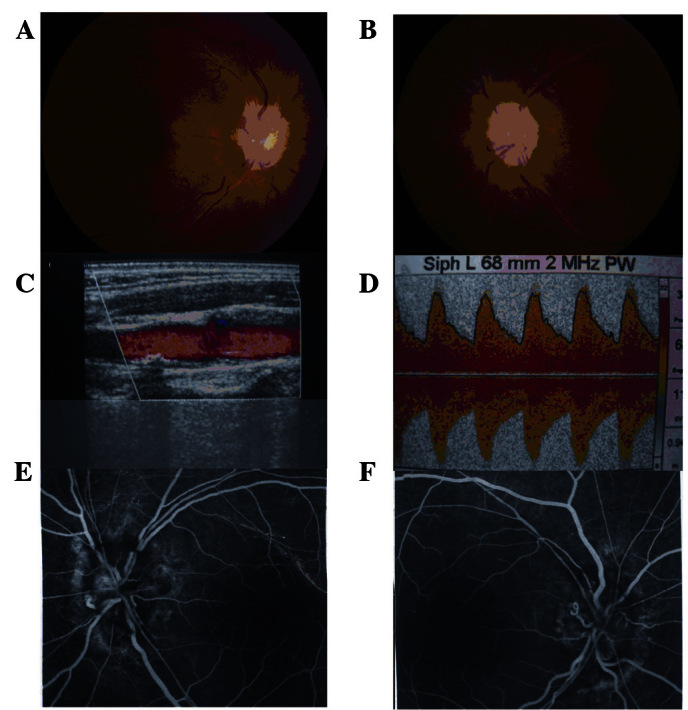
Group of images revealed ischemic optic neuropathy caused by carotid artery stenosis (female, 57 years old). (A) Fundus photograph of the right eye with ischemic optic neuropathy. (B) Fundus photograph of the left eye with ischemic optic neuropathy showed features of fundus lesions. (C) Carotid artery color Doppler ultrasound showed severe stenosis of the carotid artery and thrombogenesis in the patient. (D) Transcranial Doppler sonography showed increased resistance in the carotid siphon (indicating carotid artery stenosis). (E) FFA of the left eye showed fuzzy boundaries of the optic disc, surface telangiectasia, narrowed arteries and venous dilation. (F) FFA of the right eye showed fuzzy boundaries of optic disc, surface telangiectasia, narrowed arteries and venous dilation. FFA, fundus fluorescein angiography.

In the 8 patients who were first seen in Ophthalmology, 3 patients with ischemic optic neuropathy (Fig. 1) and 3 patients with retinal vein occlusion had been given several treatments, such as drugs to lower intraocular pressure, hypodermic injection with anisodine on the temporal side or circulation-improving medications. Two patients with neovascular glaucoma had been treated with retinal laser photocoagulation and cyclocryotherapy, respectively. The other 56 patients of group A had no eye symptoms (such as amaurosis fugax) or only had transient symptoms (such as eye swelling, periorbital pains, diplopia) so they were not diagnosed or misdiagnosed and not administered specific treatment.

### Ophthalmic artery hemodynamics indices

In the 78 patients of group A, the ophthalmic artery hemodynamics index of 34 patients was examined with color Doppler flow imaging-before and 3 months after treatment. The results suggested that all indices were significantly improved (P<0.01) after treatment with carotid artery stenting (13 cases) and carotid endarterectomy (11 cases). The indices were also improved after medical treatment (10 cases) but the difference was not statistically significant (P>0.05) (Tables III, IV and V).

### Treatment effects

Following the comprehensive analyses of patient information, the results of treatment effects are summarized in Table VI. The total effective rates of surgical and interventional treatment were higher than those observed with medical treatment (t=2.725, t=3.137, P<0.01).

## Discussion

Ocular ischemic diseases are a series of symptoms and signs, such as ocular anterior or posterior segment ischemia, that result from insufficient blood supply from the ophthalmic artery for a number of reasons (including carotid stenosis or occlusion). Ocular ischemic diseases include the following five categories: i) Ocular ischemic (hypoperfusion) syndrome. Carotid arterial atherosclerosis is the main cause, and Raynaud’s disease (also known as acral artery spasm caused by vasospastic disorder) is another possible cause. ii) Obstruction of the central retinal artery. iii) Retinal vein occlusion (ischemic type). The lumen is narrowed while the vein passes through the sieve plate area. iv) Ischemic optic neuropathy. v) Carotid cavernous fistula. Ocular ischemic diseases in the acute phase show amaurosis fugax, retinal central/branch vein occlusion, eye swelling or periorbital pains (ischemia of ocular anterior segment). As the disease develops, ocular ischemic syndrome and hypoperfusion retinopathy ultimately develop into neovascular glaucoma in the chronic phase, leading to permanent blindness (4–6).

This analysis of general information revealed that patients with ocular ischemic diseases caused by carotid artery stenosis were mostly elderly (mean age, 64.37±9.70). One-way ANOVA and unconditional logistic regression analyses also suggested that the onset of carotid artery stenosis was closely related with gender (male), hypertension, hyperlipidemia and smoking. These conclusions agreed with a previous epidemiological survey (7).

Angiography analysis showed that 75.64% (59 cases) of patients with ocular ischemic symptoms in group A had an over half diameter reduction following treatment. The degree of stenosis (mean, 69.13±7.46) was significantly higher than group B (mean, 48.34±9.23). This difference implied that ocular ischemic symptoms were one of the important signs of severe carotid artery stenosis. The majority of the 78 patients in group A were first seen in the Neurology or Neurosurgery departments. Only 8 cases (10.26%) were first diagnosed in Ophthalmology. Another 14 cases (17.95%) were seen in Ophthalmology following consultations in Neurology or Neurosurgery. The other 56 patients (71.79%) were not diagnosed or misdiagnosed, as eye symptoms (such as amaurosis fugax) were absent or only had transient symptoms (such as eye swelling, periorbital pains or diplopia). Some patients developed neovascular glaucoma and permanent blindness due to lack of timely treatment. Conversely, chief complaints including amaurosis fugax, conjunctival swelling, periorbital pains and diplopia were often misdiagnosed as asthenopia, conjunctivitis, supraorbital neuralgia and ophthalmoplegia by ophthalmologists, leading to delayed treatments.

The analyses of ocular ischemic disease composition suggested that in patients with carotid artery stenosis associated with ocular ischemic symptoms, ischemic optic neuropathy (32.26%) ranked first, followed by ocular ischemia syndrome (22.58%) and retinal central/branch vein occlusion (22.58%). External ophthalmoplegia (12.90%) and neovascular glaucoma (9.68%) ranked fourth and fifth respectively. The data provided advantageous indications for investigating risk factors of ophthalmic clinical diseases. If unexplained clinical situations, such as decreased vision, retinal central/branch vein occlusion, ocular ischemic diseases, external ophthalmoplegia or neovascular glaucoma occur, doctors should be alert to the possibility of carotid artery stenosis.

The three main treatment methods for carotid artery stenosis are medical, surgical (carotid endarterectomy) and interventional (carotid artery stenting) treatments. Therapists often provide treatments according to the degree of stenosis, age and the overall condition. Havelius *et al*(8) reported that scotopic vision and photosensitivity of patients improved significantly after carotid endarterectomy. Wolintz *et al*(9) suggested that surgical treatments were helpful for improving blood flow to the brain and eye but not for eyesight.

Most researchers (10) considered that clinical applications of carotid endarterectomy and carotid artery stenting had been used in many cases over many years, and its stable effects had been confirmed. In addition, patients treated with carotid endarterectomy or carotid artery stenting are usually provided adjuvant medical treatment, such as aspirin for anticoagulation. This comprehensive surgical treatment improves ophthalmic artery hemodynamics more effectively after carotid endarterectomy and carotid artery stenting compared with absolute medical treatment. However, there have been no large-scale clinical experiments to judge the curative effects of carotid endarterectomy or interventional treatment for eyesight improvement in China or abroad as yet (11).

This study further indicated that total effective rates of surgical and interventional treatment were higher than absolute medical treatment. Consequently, besides the necessary ophthalmic treatments, surgical and interventional treatments should also be actively applied to patients with ocular ischemic symptoms caused by carotid artery stenosis. This will help treat the primary disease and promote eye blood circulation in a timely and effective manner. In addition, the curative effects of nearly 1/5 patients were found to be invalid or uncertain after surgical or interventional treatments. This is because vision is often improved in patients with mild to moderate ocular ischemic disease but not in diseases of long duration, neovascularization disease or secondary fundus hemorrhage. Fundus lesions and visual impairment of patients with severe ocular ischemic syndromes may be irreversible, even if the ocular blood supply and flow are improved effectively. Diagnosis and treatment in a timely and correct manner is the best way to treat declining eyesight caused by carotid artery stenosis.

## Figures and Tables

**Table I:** The ocular ischemic symptoms and their frequency in group A.

Symptoms and signs	No. of cases	Percentage of cases
Amaurosis fugax	37	47.44
Eye and periorbital pains	28	35.90
Diplopia	11	14.10
Hypopsia (visual field defects)	26	33.33
Retinal hemorrhage, edema	19	24.36
Increased intraocular pressure	3	3.85

Among 78 cases, certain patients had two or more symptoms.

**Table II:** Composition of ocular ischemic diseases in group A (n=31).

Diseases	No. of cases	Constituent ratio (%)
Ischemic optic neuropathy	10	32.26
Ocular ischemia syndrome	7	22.58
Retinal central/branch vein occlusion	7	22.58
External ophthalmoplegia	4	12.90
Neovascular glaucoma	3	9.68

**Table III:** Comparison of ophthalmic artery hemodynamic indices before and after medical treatment (mean ± SD, n=10).

Index	Before treatment	After treatment	D-value	P-value
Psv (cm/sec)	18.74±5.17	27.54±6.87	8.80±5.58	0.067
Edv (cm/sec)	13.31±4.23	18.36±5.21	5.05±4.94	0.053
Vm (cm/sec)	14.57±5.03	20.17±6.07	5.60±6.16	0.057
RI	0.8±0.14	0.43±0.11	0.41±0.21	0.058
PI	1.48±0.47	0.81±0.29	0.67±0.38	0.063

Psv, peak systolic velocity; Edv, end diastolic velocity; Vm, mean glow velocity; RI, resistive index; PI, pulsatility index.

**Table IV:** Comparison of ophthalmic artery hemodynamic indices before and after surgical treatment (mean ± SD, n=11).

Index	Before treatment	After treatment	D-value	P-value
Psv (cm/sec)	9.12±2.28	28.58±3.63	19.46±4.01	<0.01
Edv (cm/sec)	7.03±1.35	20.83±3.12	13.80±2.42	<0.01
Vm (cm/sec)	8.09±1.85	25.83±3.56	17.74±3.31	<0.01
RI	1.17±0.06	0.33±0.09	0.84±0.03	<0.01
PI	1.69±0.29	0.61±0.13	1.08±0.32	<0.01

Psv, peak systolic velocity; Edv, end diastolic velocity; Vm, mean glow velocity; RI, resistive index; PI, pulsatility index.

**Table V:** Comparison of ophthalmic artery hemodynamic indices before and after interventional treatment (mean ± SD, n=13).

Index	Before treatment	After treatment	D-value	P-value
Psv (cm/sec)	8.32±2.28	24.91±3.76	16.59±3.91	<0.01
Edv (cm/sec)	7.17±1.35	20.72±2.92	13.55±2.24	<0.01
Vm (cm/sec)	8.76±1.85	25.30±3.73	16.56±2.93	<0.01
RI	1.28±0.06	0.52±0.14	0.76±0.11	<0.01
PI	1.58±0.29	0.61±0.23	0.97±0.36	<0.01

Psv, peak systolic velocity; Edv, end diastolic velocity; Vm, mean glow velocity; RI, resistive index; PI, pulsatility index.

**Table VI:** Comparison of effects of three different treatment methods.

Treatment	Significantly effective	Improved	Invalid	Uncertain	Total effective rate(%)
Medical	3	5	8	3	42.11
Interventional	12	9	3	2	80.77
Surgical	17	11	3	2	84.85
